# Supplementary material for: Development and Evaluation of a Robust Sandwich Immunoassay System Detecting Serum WFA-Reactive IgA1 for Diagnosis of IgA Nephropathy
Source: Int J Mol Sci. 2022 May 5;23(9):5165. doi: 10.3390/ijms23095165 (PMC9104065; doi:10.3390/ijms23095165)
Supplement: Supplementary file 1 [file ijms-23-05165-s001.zip › ijms-1681210-supplementary.pdf]

Supporting Materials

Development and Evaluation of a Robust Sandwich Immunoassay System Detecting Serum WFA-Reactive IgA1 for Diagnosis of IgA Nephropathy

Yuta Uenoyama [1,†], Atsushi Matsuda [1,†], Kazune Ohashi [1], Koji Ueda [1], Misaki Yokoyama [1], Takuya Kyoutou [1], Kouji Kishi [2], Youichi Takahama [1], Masaaki Nagai [3], Takaaki Ohbayashi [3], Osamu Hotta [4], and Hideki Matsuzaki [1,*]

Supplementary Figure S1. Layout of 45 lectins immobilized on a LecChip™ microarray slide.

The properties of each lectin are shown in Supplementary Table S1.

Supplementary Figure S2. Lectin microarray analysis of purified IgA1 from an IgAN patient.

The purified IgA1 from an IgAN patient was treated with PNGase before lectin array analysis performed with or without N-glycan digestion. A. Signal pattern of 45 lectins in PNGase (+) or (-) of purified IgA1. B. WFA signals among the signal pattern of A.

Supplementary Figure S3. Lectin microarray analysis of native IgA1 standard with or without sialic acid digestion by sialidase.

Native IgA1 was directly fluorescent labeled. After labeling, sialidase treatment and lectin microarray analysis were performed.

Supplementary Figure S4. Lectin microarray analysis of constructed agglutinated IgA1 standard.

Analysis of Glt-IgA1 was performed by antibody-overlay lectin microarray. Comparison of lectin microarray analysis between nIgA1 without sialidase treatment and Glt-IgA1 with or without sialidase treatment. WFA signals among the signal pattern of A are shown in Figure 3C.

Supplementary Figure S5. Transitions of serum WFA+-IgA1 levels according to follow-up periods after tonsillectomy.

Supplementary Figure S6. Comparison of other IgAN markers, Gd-IgA1, total IgA, and C3 levels to distinguish IgAN from non-IgAN and NCs.

Dot plots of serum levels of Gd-IgA, total IgA, and C3 measured using the same cohort as in Figure 4. The red bar indicates the median of each measurement. $^{**}P < 0.01$, $^{*}P < 0.05$.

Supplementary Figure S7. Correlation of WFA+-IgA1 and other IgAN markers.
2D plots of the correlation between Serum WFA+-IgA1 and Gd-IgA1, total IgA, C3 in 50 NCs, 43 non-IgAN, and 47 IgAN patients. Blue, green, and red indicate NC, non-IgAN, and IgAN patients, respectively.

Supplementary Figure S8. ROC curve analysis of each marker, and combination of each marker and WFA+-IgA1.

Receiver operating curve (ROC) curve analysis was performed to distinguish 47 IgAN from 50 NCs and 43 non-IgAN. A. These panels show ROC analysis results with a single marker. B. These panels are the ROC analysis results in the combination analysis.

Supplementary Figure S9. Glt-IgA1 chromatogram with SEC for sorting.

Supplementary Figure S10. Workflow of detection process with HISCL automated CLEIA analyzer

Supplementary Table S1. Abbreviations and carbohydrate specificities of 45 lectins of lectin microarray.

Data are compiled from the Lectin Frontier Database (LfDB; http://riodb.ibase.aist.go.jp/rcmg/glycodb/LectinSerch)

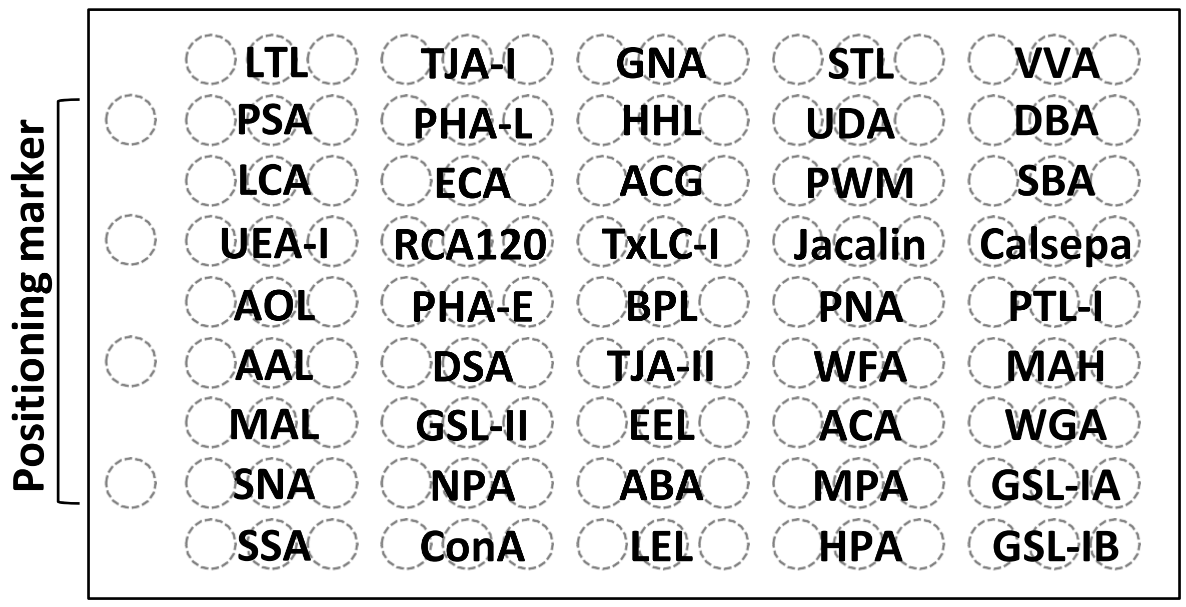

Supplementary Figure S1. Layout of 45 lectins immobilized on a LecChip™ microarray slide.

The properties of each lectin are shown in Supplementary Table S1.

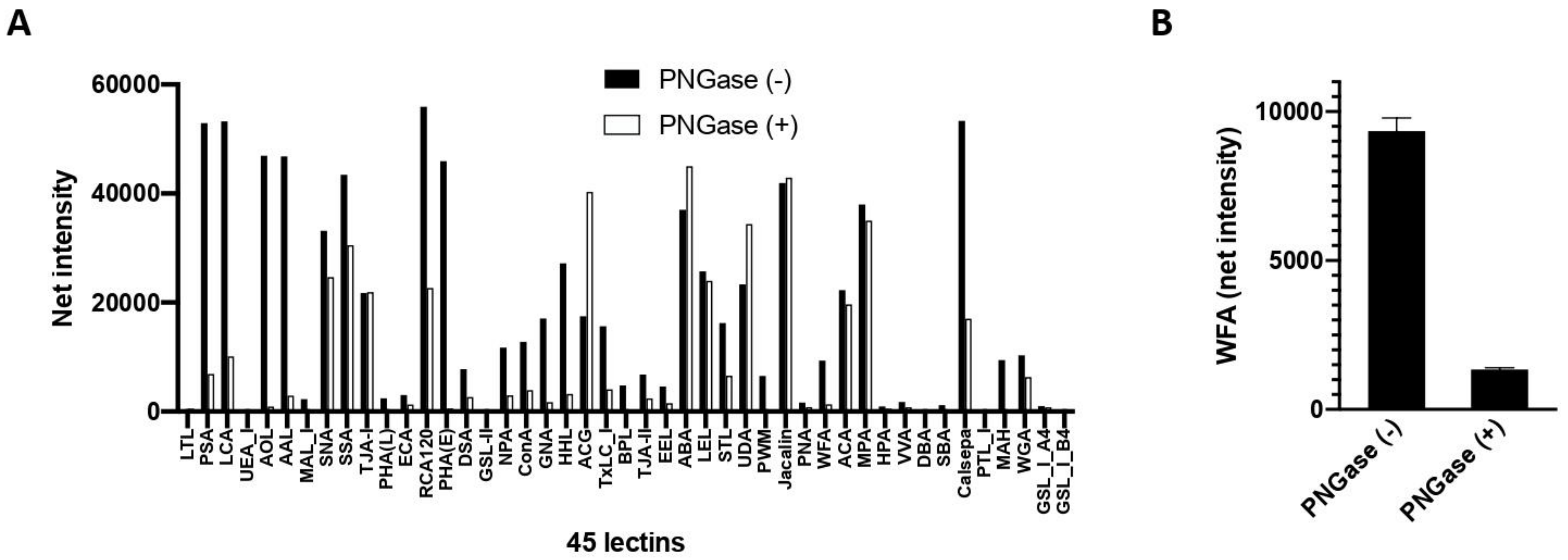

Supplementary Figure S2. Lectin microarray analysis of purified IgA1 from an IgAN patient.

The purified IgA1 from an IgAN patient was treated with PNGase before lectin array analysis performed with or without N-glycan digestion. A. Signal pattern of 45 lectins in PNGase (+) or (-) of purified IgA1. B. WFA signals among the signal pattern of A.

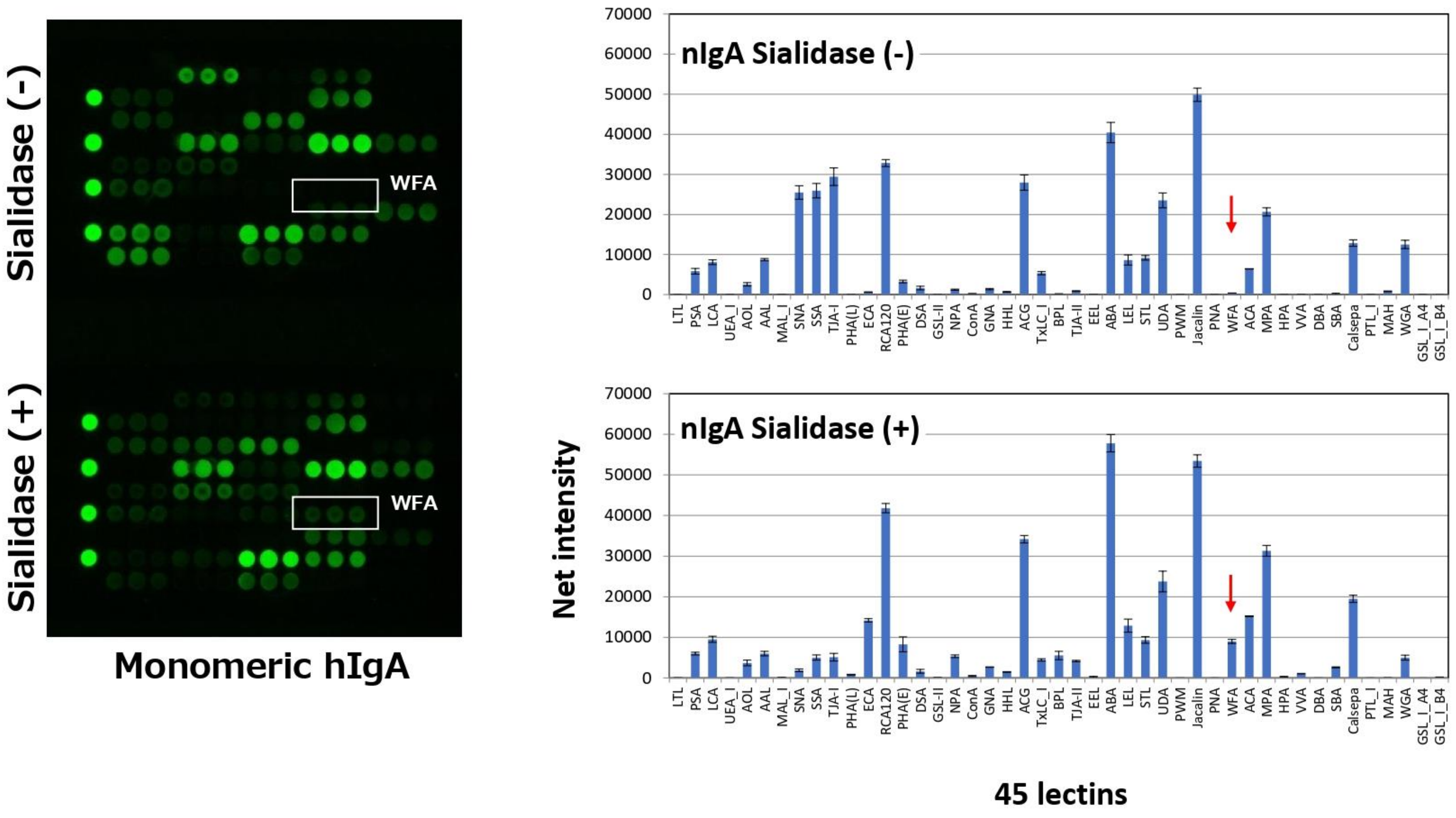

Supplementary Figure S3. Lectin microarray analysis of native IgA1 standard with or without sialic acid digestion by sialidase.

Native IgA1 was directly fluorescent labeled. After labeling, sialidase treatment and lectin microarray analysis were performed.

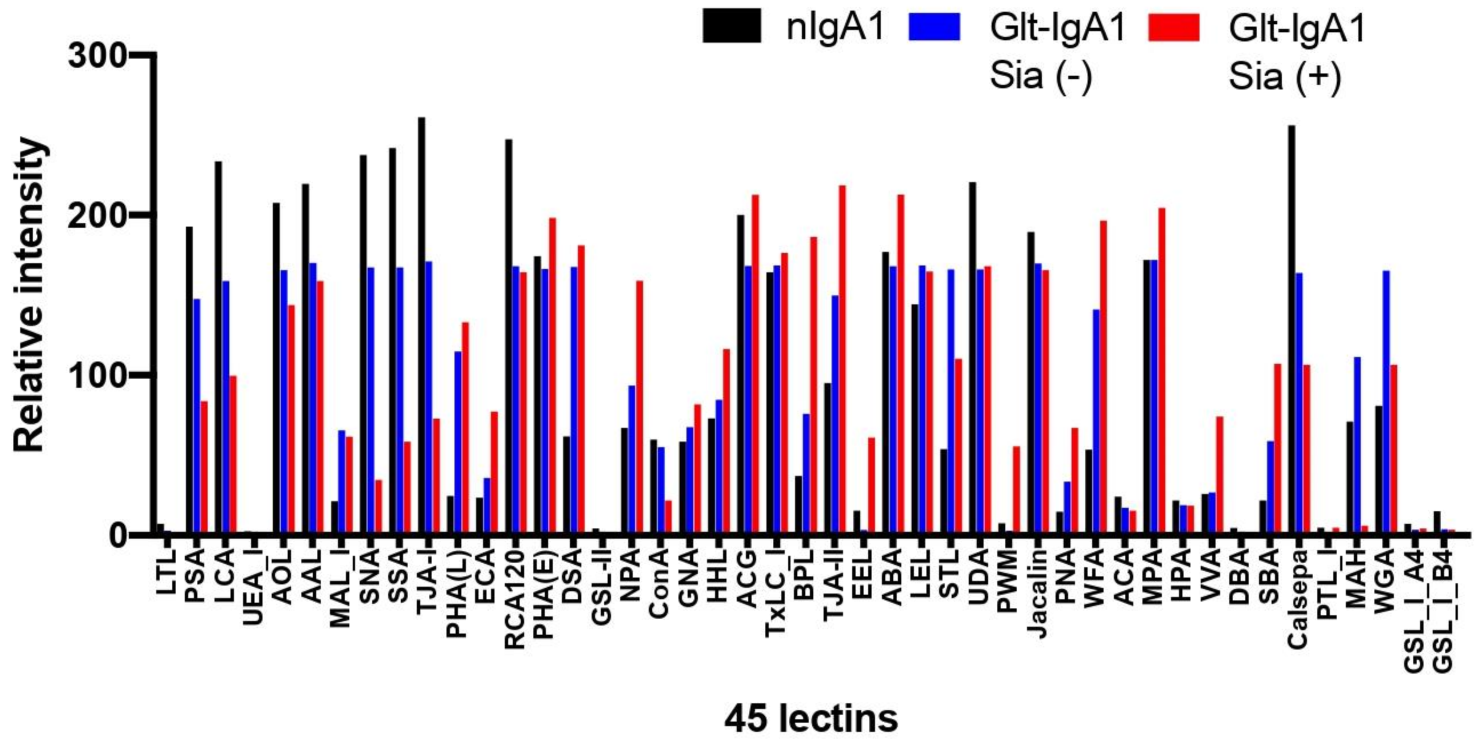

Supplementary Figure S4. Lectin microarray analysis of constructed agglutinated IgA1 standard.

Analysis of Glt-IgA1 was performed by antibody-overlay lectin microarray. Comparison of lectin microarray analysis between nIgA1 without sialidase treatment and Glt-IgA1 with or without sialidase treatment. WFA signals among the signal pattern of A are shown in Figure 3C.

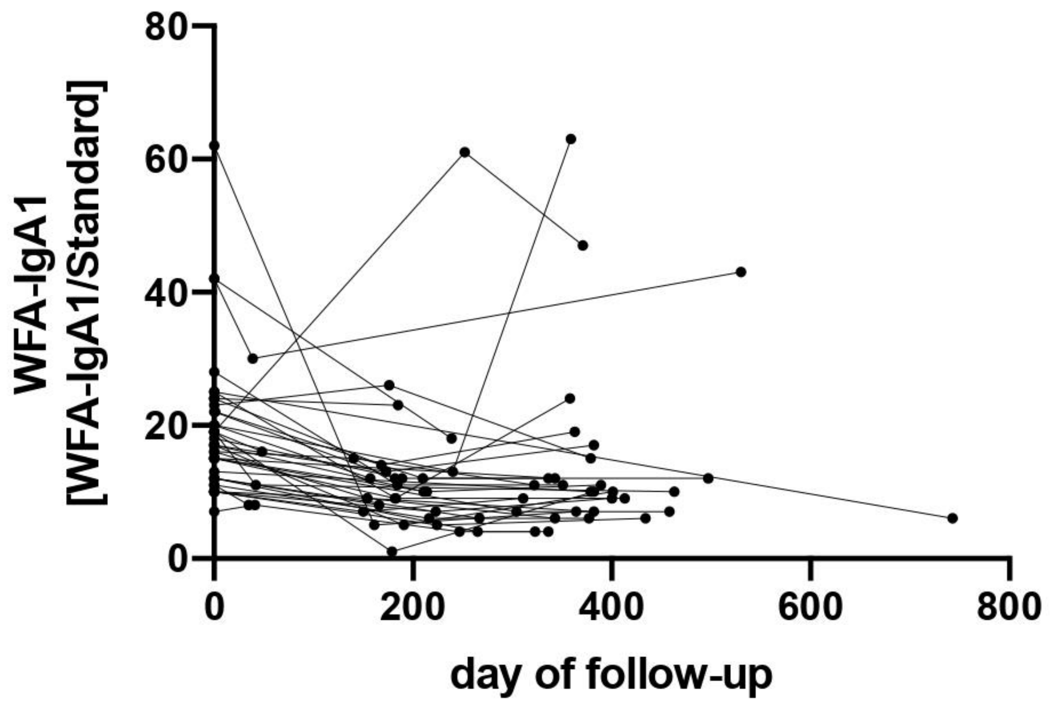

Supplementary Figure S5. Transitions of serum WFA+-IgA1 levels according to follow-up periods after tonsillectomy.

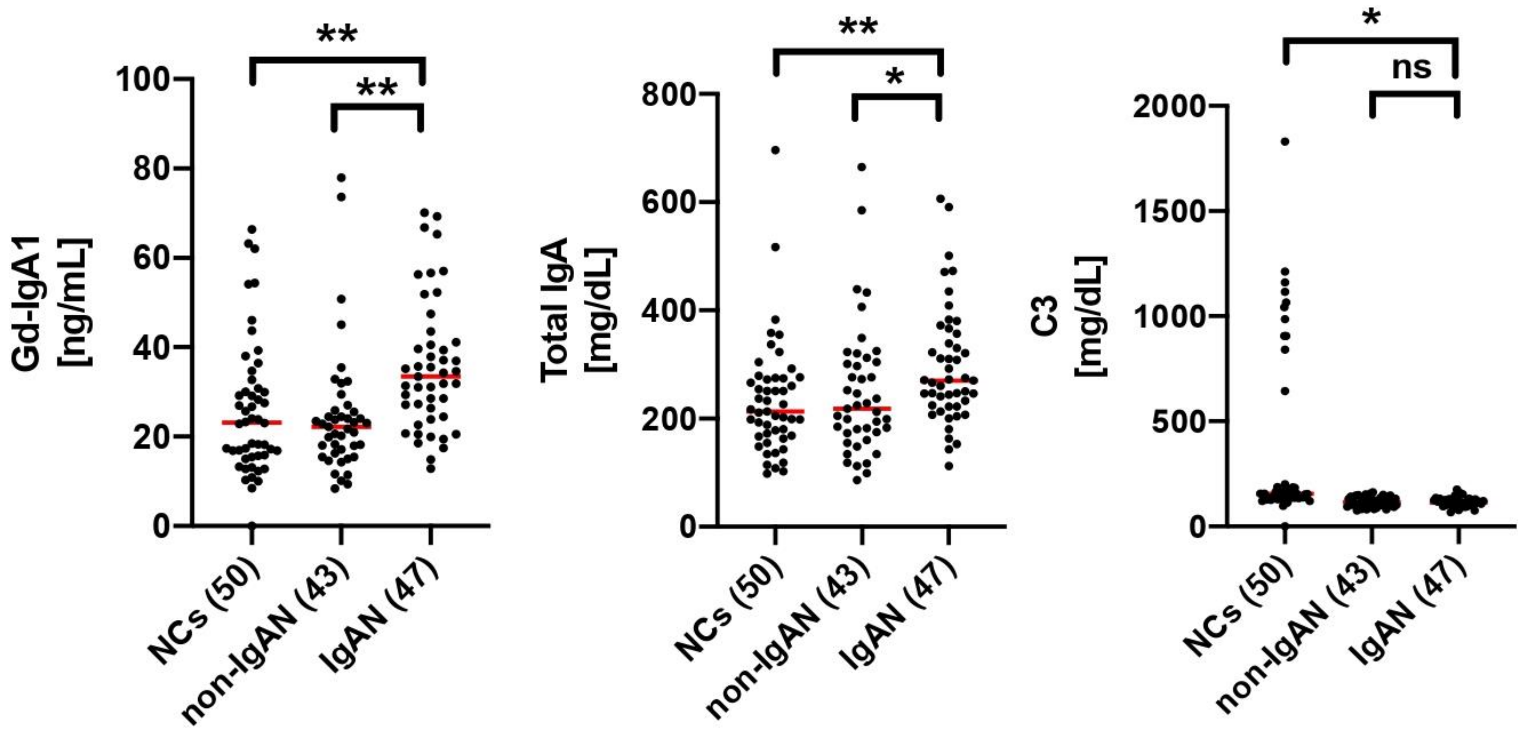

Supplementary Figure S6. Comparison of other IgAN markers, Gd-IgA1, total IgA, and C3 levels to distinguish IgAN from non-IgAN and NCs.

Dot plots of serum levels of Gd-IgA, total IgA, and C3 measured using the same cohort as in Figure 4. The red bar indicates the median of each measurement.
$**P < 0.01$, $*P < 0.05$.

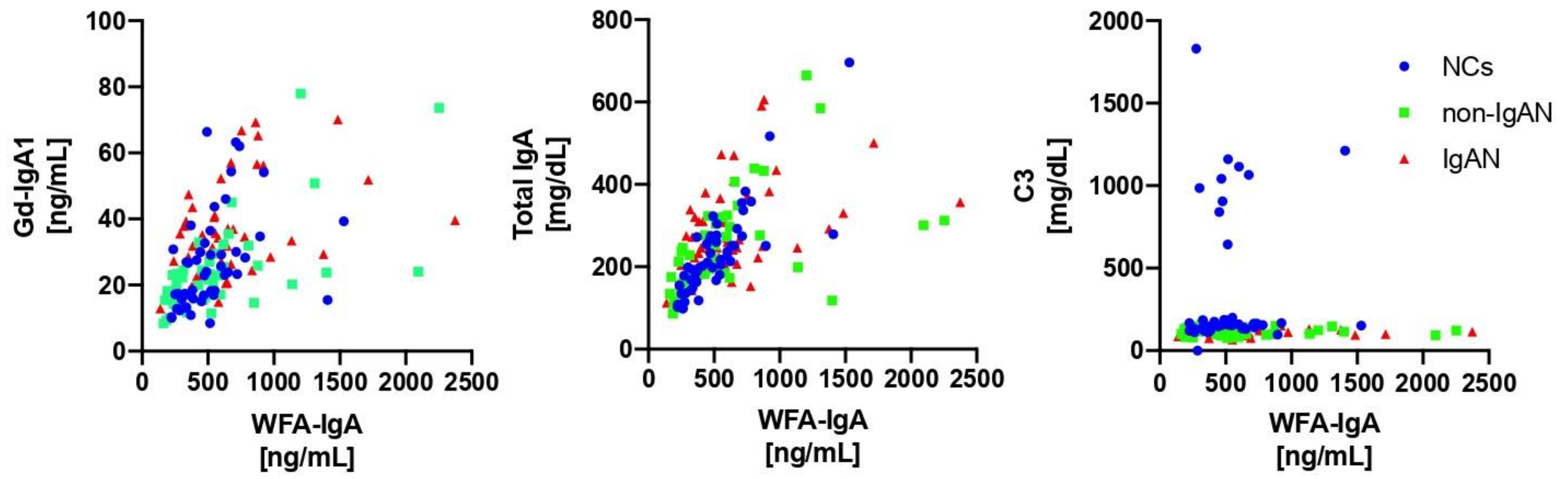

Supplementary Figure S7. Correlation of WFA+-IgA1 and other IgAN markers. 2D plots of the correlation between Serum WFA+-IgA1 and Gd-IgA1, total IgA, C3 in 50 NCs, 43 non-IgAN, and 47 IgAN patients. Blue, green, and red indicate NC, non-IgAN, and IgAN patients, respectively.

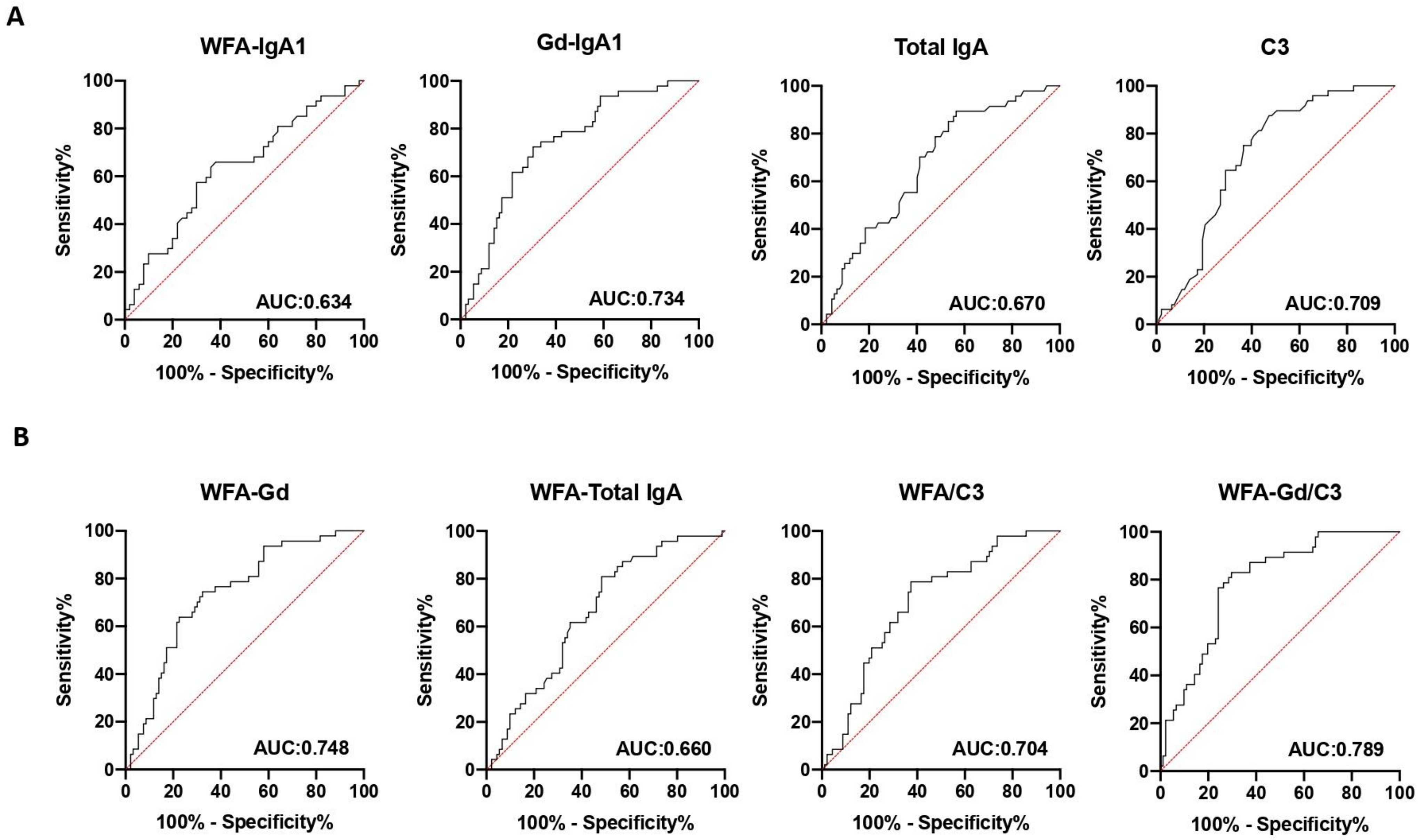

Supplementary Figure S8. ROC analysis of each single marker and combination of each marker andWFA+-IgA1.

Receiver operating curve (ROC) curve analysis was performed to distinguish 47 IgAN from 50 NCs and 43 non-IgAN. A. These panels show ROC analysis results with a single marker. B. These panels are the ROC analysis results in the combination analysis.

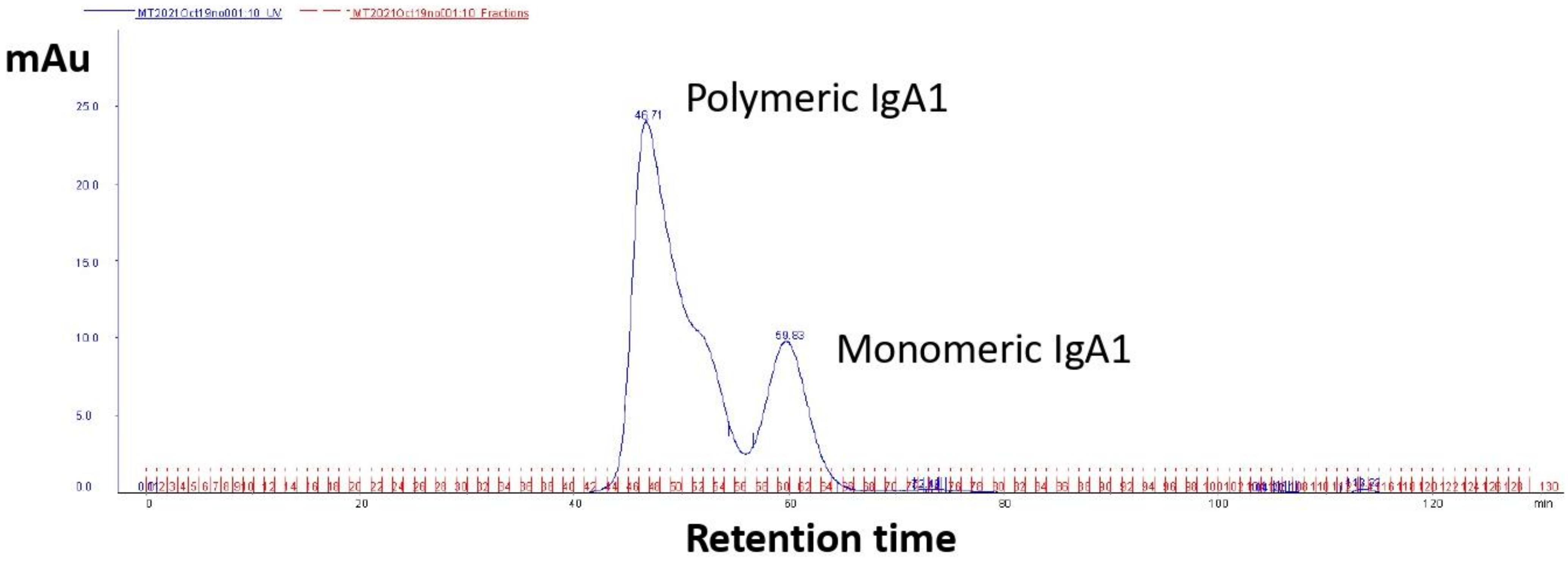

Supplementary Figure S9. Glt-IgA1 chromatogram with SEC for sorting.

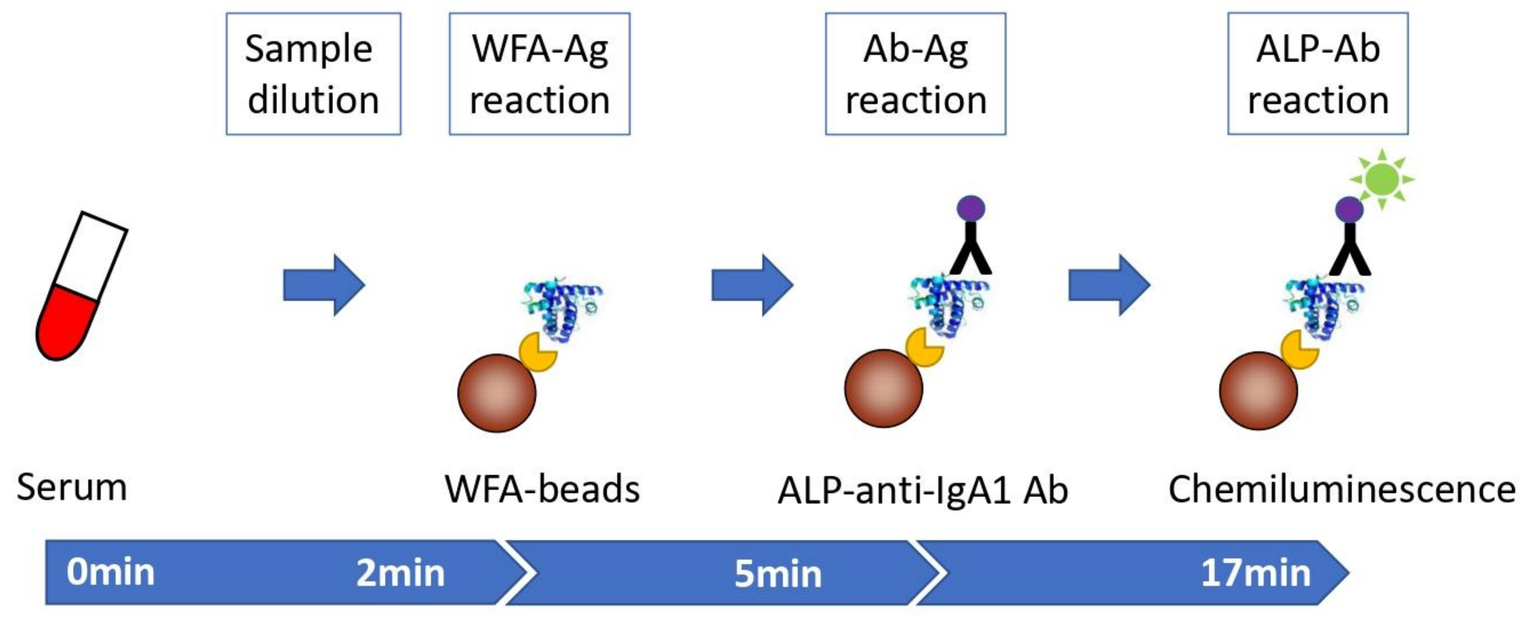

Supplementary Figure S10. Workflow of detection process with the HISCL CLEIA analyzer.

Supplementary Table S1. Abbreviations and carbohydrate specificities of 45 lectins in the lectin microarray. Data are compiled from the Lectin Frontier Database (LfDB; http://riodb.ibase.aist.go.jp/rcmg/glycodb/LectinSerch)

	Lectins	Origin	Binding specificity
1	LTL	*Lotus tetragonobus*	Fucα1-3GlcNAc, Sia-Le^x and Le^x
2	PSA	*Pisum sativum*	Fucα1-6GlcNAc and α-Man
3	LCA	*Lens culinaris*	Fucα1-6GlcNAc and α-Man , α-Glc
4	UEA-I	*Ulex europaeus*	Fucα1-2LacNAc
5	AOL	*Aspergillus oryzae*	Terminal α-Fuc, Sia-Le^x and Le^x
6	AAL	*Aleuria aurantia*	Terminal α-Fuc, Sia-Le^x and Le^x
7	MAL	*Maackia amurensis*	Siaα2-3Gal
8	SNA	*Sambucus nigra*	Siaα2-6Gal/GalNAc
9	SSA	*Sambucus sieboldiana*	Siaα2-6Gal/GalNAc
10	TJA-I	*Trichosanthes japonica*	Siaα2-6Galβ1-4GlcNAcβ-R
11	PHA-L	*Phaseolus vulgaris*	Tri-and tetra-antennary complex oligosaccharides
12	ECA	*Erythrina cristagalli*	Lac/LacNAc
13	RCA120	*Ricinus communis*	Lac/LacNAc
14	PHA-E	*Phaseolus vulgaris*	NA_2 and bisecting GlcNAc
15	DSA	*Datura stramonium*	$(GlcNAc)_n$, polyLacNAc and LacNAc (NA_3, NA_4)
16	GSL-II	*Griffonia simplicufolia*	Agalactosylated N-glycan
17	NPA	*Narcissus pseudonarcissus*	Non-substituted α1-6Man
18	ConA	*Canavalia ensiformis*	α1-6Man (inhibited by presence of bisecting GlcNAc)
19	GNA	*Galanthus nivalis*	Non-substituted α1-6Man
20	HHL	*Hippeastrum Hybrid*	Non-substituted α1-6Man
21	ACG	*Agrocybe cylindracea*	Siaα2-3Galβ1-4 GlcNAc
22	TxLC-I	*Tulipa gesneriana*	Man3 core, bi- and tri-antenary complex-type N-Glycan, GalNAc
23	BPL	*Bauhinia purpurea*	Galβ1-3GalNAc and NA_3, NA4
24	TJA-II	*Trichosanthes japonica*	Fucα1-2Gal, β-GalNAc > NA3, NA4
25	EEL	*Euonymus europaeus*	Galα1-3[Fuc α1-2 Gal] > Gal α1-3 Gal
26	ABA	*Agaricus bisporus*	Galβ1-3GalNAcα-Thr/Ser (T) and sialyl-T
27	LEL	*Lycipersicon esculentum*	(GlcNAc)n and polyLacNAc
28	STL	*Solanum tuberosum*	(GlcNAc)n and polyLacNAc
29	UDA	*Urtica dioica*	(GlcNAc)n and polyLacNAc
30	PWM	*Phytolacca americana*	(GlcNAc)n and polyLacNAc
31	Jacalin	*Artocarpas integliforia*	Galβ1-3GalNAcα-Thr/Ser (T) and GalNAcα-Thr/Ser (Tn)
32	PNA	*Arachis hypogaea*	Galβ1-3GalNAcα-Thr/Ser (T)
33	WFA	*Wisteria floribunda*	Terminal GalNAc (e.g., GalNAcβ1-4GlcNAc)
34	ACA	*Amaranthus caudatus*	Galβ1-3GalNAcα-Thr/Ser (T)
35	MPA	*Maclura pomifera*	Galβ1-3GalNAcα-Thr/Ser (T) and GalNAcα-Thr/Ser (Tn)
36	HPA	*Helix pomatia*	Terminal GalNAc
37	VVA	*Vicia villosa*	α-, β-linked terminal GalNAc and GalNAcα-Thr/Ser (Tn)
38	DBA	*Dolichos biflorus*	GalNAcα-Thr/Ser (Tn) and GalNAcα1-3GalNAc
39	SBA	*Glycine max*	Terminal GalNAc (especially GalNAcα1-3Gal)
40	Calsepa	*Calystegia sepium*	Man, Maltose
41	PTL-I	*Psophocarpus tetragonolobus*	α-GalNAc and Gal
42	MAH	*Maackia amurensis*	Siaα2-3Galβ1-3[Siaα2-6GalNAc]α-R
43	WGA	*Triticum aestivum*	(GlcNAc)n and multivalent Sia
44	GSL-IA4	*Griffonia Simplicifolia*	α-GalNAc, GalNAcα-Thr/Ser (Tn)
45	GSL-IB4	*Griffonia Simplicifolia*	α-Gal
